# Effects of icosapent ethyl on lipid and inflammatory parameters in patients with diabetes mellitus-2, residual elevated triglycerides (200–500 mg/dL), and on statin therapy at LDL-C goal: the ANCHOR study

**DOI:** 10.1186/1475-2840-12-100

**Published:** 2013-07-09

**Authors:** Eliot A Brinton, Christie M Ballantyne, Harold E Bays, John J Kastelein, Rene A Braeckman, Paresh N Soni

**Affiliations:** 1 Utah Foundation for Biomedical Research, Salt Lake City, UT, USA; 2 Baylor College of Medicine and the Methodist DeBakey Heart and Vascular Center, Houston, TX, USA; 3 Louisville Metabolic and Atherosclerosis Research Center, Louisville, KY, USA; 4 Academic Medical Center, Amsterdam, The Netherlands; 5 Amarin Pharma Inc., Bedminster, NJ, USA; 6 Amarin Pharma Inc., Groton, CT, USA

**Keywords:** Apolipoprotein B, Cholesterol, Diabetes mellitus, Eicosapentaenoic acid, Hydroxymethylglutaryl-CoA reductase inhibitors, Hypertriglyceridemia, Icosapent ethyl, Low density lipoprotein, Triglycerides

## Abstract

**Background:**

Icosapent ethyl (IPE) is a high-purity prescription form of eicosapentaenoic acid (EPA) ethyl ester indicated as an adjunct to diet to reduce triglyceride (TG) levels in adult patients with severe (≥500 mg/dL) hypertriglyceridemia. ANCHOR was a 12-week, phase 3 study that evaluated the efficacy and safety of IPE in patients (N = 702) with residual high fasting TG levels (≥200 and <500 mg/dL) despite having optimized low-density lipoprotein cholesterol (LDL-C) levels (≥40 and <100 mg/dL) on statin therapy. Among patients randomized to IPE (4 g/day or 2 g/day) or placebo, 514 (73%) had diabetes mellitus.

**Methods:**

A post hoc subgroup analysis of the ANCHOR study was conducted to assess the effects of IPE on median placebo-adjusted percent change from baseline in efficacy end point parameters in 3 subgroups: total (all subjects with diabetes—overall median baseline glycosylated hemoglobin A_1c_ [A_1c_] = 6.8%), better-controlled diabetes (below median baseline A_1c_), and less-controlled diabetes (above median baseline A_1c_).

**Results:**

Baseline efficacy parameters were similar among all groups except high-sensitivity C-reactive protein (hsCRP), which was higher in the total and less-controlled diabetes groups. Compared with placebo, IPE 4 g/day significantly reduced TG, non-high-density lipoprotein cholesterol, very-low-density lipoprotein cholesterol (VLDL-C), lipoprotein-associated phospholipase A_2_, apolipoprotein B (Apo B), total cholesterol, high-density lipoprotein cholesterol, VLDL-TG, oxidized LDL, and remnant-like particle cholesterol in all 3 diabetes groups, LDL-C in the total diabetes group, and hsCRP in the total and less-controlled diabetes groups. Decreases in hsCRP and Apo B were much greater in patients with less-controlled diabetes. There were no significant increases in fasting plasma glucose, A_1c_, insulin, or homeostasis model assessment-estimated insulin resistance in any group.

**Conclusion:**

IPE 4 g/day significantly improved lipid and lipid-related parameters without worsening glycemic control in patients with diabetes and mixed dyslipidemia, with possibly greater effects among those with less-controlled diabetes.

**Trial registration:**

Clinicaltrials.gov Identifier NCT01047501

## Introduction

The US Center for Disease Control and Prevention reports that the crude age-adjusted prevalence of diagnosed diabetes mellitus has risen by >175% from 1980 to 2010 [1]. The risk for all forms of cardiovascular disease (CVD) is considerably elevated in patients with diabetes mellitus, and this is driven in part by the high prevalence of dyslipidemia in this population [2]. Low-density lipoprotein cholesterol (LDL-C) levels ≥100 mg/dL are found in approximately 64% of adults with diabetes mellitus-2, while 35% have fasting triglyceride (TG) levels ≥200 mg/dL [3]. Elevated TG levels in people with diabetes mellitus are often associated with decreased high-density lipoprotein cholesterol (HDL-C) and small, dense LDL particles [4]. Although reducing LDL-C with statins is the basis of dyslipidemia treatment in patients with diabetes mellitus, considerable residual CVD risk remains in statin-treated patients [5,6]. Non–HDL-C is the secondary treatment target if TG levels remain ≥200 and <500 mg/dL after optimization of statin therapy [7,8]. Modifying other lipid, lipoprotein, and inflammation-related risk factors beyond LDL-C and non–HDL-C may provide additional approaches in addressing the residual CVD risk [5].

Omega-3 (OM-3) fatty acids have the potential to lower CVD risk, as they have been shown to improve several cardiovascular risk factors, particularly lowering TG levels [4,9]. Currently approved OM-3 fatty acid formulations contain either a combination of eicosapentaenoic acid (EPA) and docosahexaenoic acid (DHA), or EPA alone. Icosapent ethyl (IPE; Vascepa® [formerly AMR101]; Amarin Pharma Inc., Bedminster, NJ, USA) is a high-purity prescription form of EPA ethyl ester approved by the US Food and Drug Administration as an adjunct to diet to reduce TG levels in adult patients with severe (≥500 mg/dL) hypertriglyceridemia.

In the 12-week ANCHOR study of patients with residual high TG levels (200–500 mg/dL) despite having optimized LDL-C (40–100 mg/dL) while on statin therapy, IPE 4 g/day was shown to significantly reduce TG, LDL-C, non–HDL-C, very-low-density lipoprotein cholesterol (VLDL-C), lipoprotein-associated phospholipase A_2_ (Lp-PLA_2_), apolipoprotein B (Apo B), total cholesterol (TC), HDL-C, VLDL-TG, and high-sensitivity C-reactive protein (hsCRP) levels compared with placebo [10]. IPE was generally well tolerated, with a safety and tolerability profile similar to placebo. A high percentage (73%) of the ANCHOR study patients had diabetes mellitus. The objective of this post hoc subgroup analysis was to evaluate the effects of IPE on lipids, lipoproteins, and inflammatory biomarkers in the patients with diabetes mellitus from the ANCHOR study, with subanalysis by degree of glycemic control (glycosylated hemoglobin A_1c_ [A_1c_] above or below the median).

## Methods

The cohort of patients with diabetes mellitus in the ANCHOR study was selected from the overall study population. The methods used in ANCHOR have been published previously [10]. Briefly, ANCHOR was a 12-week, phase 3, multicenter, double-blind study including patients >18 years of age at high risk for CVD (patients with clinical coronary heart disease [CHD] or CHD risk equivalents [10-year risk ≥20%]) as defined by the National Cholesterol Education Program Adult Treatment Panel III (NCEP-ATP III) guidelines [11]. The protocol was approved by the appropriate international review boards, and all patients underwent the informed consent process prior to enrollment, as evidenced by their written informed consent. Patients were required to have been on stable statin therapy (atorvastatin, rosuvastatin, or simvastatin with or without ezetimibe) for ≥4 weeks at doses expected to produce “optimal” LDL-C levels for high-risk patients (≥40 and <100 mg/dL) and to continue the statin therapy throughout the study [10,11]. Patients were also required to maintain a stable diet and level of exercise during the study [10].

Once enrolled, patients entered a 4- to 6-week lead-in diet and lifestyle stabilization period. This lead-in period also served as a medication washout period for any patients taking non-statin lipid-altering medications such as niacin >200 mg daily, fibrates, fish oil, other products containing OM-3 fatty acids, or other herbal products or dietary supplements with potential lipid-altering effects at the time of screening (6-week washout was required for such patients). A 2- to 3-week lipid-qualifying period followed the lead-in period, during which the specified criteria of TG levels ≥200 and <500 mg/dL and LDL-C ≥40 and <100 mg/dL were required to be met. Patients who met the lipid-qualifying criteria were randomized to IPE 4 g/day, 2 g/day, or matching placebo and entered the double-blind, 12-week safety and efficacy period.

The presence of diabetes mellitus type 1 or 2 was considered to be one of the criteria for meeting the inclusion requirement of the presence of CHD risk equivalents as defined by the NCEP-ATP III guidelines [11]. Patients who had A_1c_ >9.5% or were being treated with antidiabetes medication that had not been stable for ≥4 weeks at screening were excluded from the ANCHOR study.

All of the diabetes subgroup analyses assessing the effects of IPE on lipids, lipoproteins, and biomarkers of inflammation were performed post hoc, with the exception of TG level analysis, which was prespecified. The effects of IPE on lipid, lipoprotein, and inflammation biomarker parameters were assessed in the intent-to-treat (ITT) population, defined as all randomized patients who took ≥1 dose of any study drug, had a valid baseline laboratory measurement, and had ≥1 valid post-randomization laboratory efficacy measurements of any type (ie, lipid, lipoprotein, and inflammatory parameters). The efficacy analysis was examined in 3 subgroups: total (all subjects with diabetes mellitus—overall median baseline A_1c_ = 6.8%), patients with better-controlled diabetes mellitus (percent A_1c_ < overall median A_1c_), and patients with less-controlled diabetes mellitus (percent A_1c_ ≥ overall median A_1c_). Subjects with missing baseline or week 12 measurements, because of either missing laboratory samples or unreportable values, were excluded from the analysis.

The primary efficacy variable was the median placebo-adjusted percent change from baseline to week 12 in fasting TG levels. Secondary efficacy variables included LDL-C, non–HDL-C, VLDL-C, Lp-PLA_2_, and Apo B. Exploratory efficacy variables included TC, HDL-C, VLDL-TG, hsCRP, oxidized LDL (Ox-LDL), and remnant-like particle cholesterol (RLP-C). Exploratory diabetes parameters included fasting plasma glucose (FPG), A_1c_, and homeostasis model assessment-estimated insulin resistance (HOMA-IR). Laboratory measurements were performed by the central laboratory, Medpace Reference Laboratories (Cincinnati, Ohio) as follows: Serum TG and cholesterol concentrations were measured using enzymatic colorimetric tests (Olympus AU2700 or AU5400 Analyzer, Olympus, Center Valley, PA, USA) with calibration directly traceable to the Centers for Disease Control reference procedures. Serum HDL-C was isolated by precipitating Apo B–containing lipoproteins (chylomicrons, VLDLs, intermediate-density lipoproteins, and LDLs) with dextran sulfate, and HDL-C was measured in the supernatant [12]. Non–HDL-C was calculated by subtracting HDL-C from TC. Serum LDL-C, VLDL-C, and VLDL-TG were calculated from TC and TG and measured by beta-quantification after preparative ultracentrifugation [13]. Serum Apo B was measured using rate immunonephelometry (Dade Behring BNII nephelometer, Siemens Healthcare Diagnostics, Deerfield, IL, USA). Immunonephelometry was used to measure serum hsCRP (Dade Behring BNII nephelometer), serum RLP-C was measured with an immunoseparation assay by Polymedco (Cortlandt Manor, NY, USA) on the Daytona chemistry analyzer (Randox, Crumlin, United Kingdom), and plasma Ox-LDL concentrations were measured with solid-phase 2-site enzyme immunoassay (Mercodia, Winston Salem, NC, USA). A_1c_, FPG, and insulin were measured with high-performance liquid chromatography, photometry, and an electrochemiluminescence immunoassay, respectively. HOMA-IR was calculated as described by Matthews et al. [14]. Berkeley Heart Laboratory (Burlingame, CA, USA) measured Lp-PLA_2_ using the PLAC® enzyme-linked immunosorbent assay (diaDexus, South San Francisco, CA, USA).

Descriptive statistics and analyses were applied to the diabetes subgroups, which were similar to those performed on the overall ANCHOR study ITT population [10]. Because significant departures from normality were observed for the overall ITT population, a nonparametric analysis was performed for the subgroups, and medians and quartiles were provided for each treatment group. The median difference of each variable (percent change from baseline) between each IPE treatment group and the placebo group was evaluated with a nonparametric test using the Hodges-Lehmann medians of the differences between treatment groups and the Wilcoxon rank-sum test. For exploratory efficacy parameters, it was prespecified that no adjustments were to be made for multiplicity and that significance was defined as a *P* ≤ 0.05.

## Results

Among the patients randomized in the ANCHOR study, 513 (73%) were determined to have diabetes mellitus type 2. One patient had diabetes mellitus type 1. In the ITT population (n = 501), there were 165, 171, and 165 patients with diabetes mellitus assigned to the IPE 4 g/day, 2 g/day, and placebo groups, respectively. The overall median baseline A_1c_ was 6.8% for the randomized population of patients with diabetes mellitus. In the subgroup of patients with baseline A_1c_ values below the overall median baseline value (ie, better-controlled diabetes), the mean baseline A_1c_ was 6.2% (n = 253). In the subgroup of patients with A_1c_ values above the median baseline value (ie, less-controlled diabetes), the mean baseline A_1c_ was 7.6% (n = 261).

**Figure 1:**
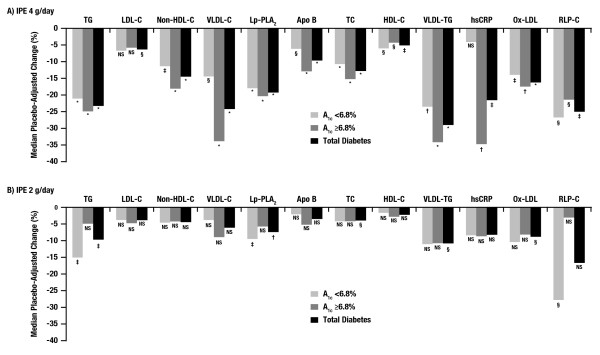
**Median placebo-adjusted percent change from baseline to week 12 for efficacy end points in diabetes subgroups by median baseline A**_**1c**_**. A)** IPE 4 g/day; **B)** IPE 2 g/day. *P ≤ 0.0001; ^†^*P* < 0.001; ^‡^*P* < 0.01; ^§^*P* ≤ 0.05. A_1c_ = glycosylated hemoglobin A_1c_; Apo B = apolipoprotein B; HDL-C = high-density lipoprotein cholesterol; hsCRP = high-sensitivity C-reactive protein; IPE = icosapent ethyl; LDL-C = low-density lipoprotein cholesterol; Lp-PLA_2_ = lipoprotein-associated phospholipase A_2_; non–HDL-C = non-high-density lipoprotein cholesterol; NS = not significant; Ox-LDL = oxidized low-density lipoprotein; RLP-C = remnant-like particle cholesterol; TC = total cholesterol; TG = triglyceride; VLDL-C = very-low-density lipoprotein cholesterol; VLDL-TG = very-low-density lipoprotein triglycerides.

Baseline values for the efficacy end points are shown in Table 1. In the IPE 4 g/day group, baseline values were similar between all diabetes subgroups for all lipid, lipoprotein, and inflammatory parameters with the exception of hsCRP, which was numerically higher in the less-controlled diabetes subgroup than in the better-controlled diabetes subgroup. Similar results were observed in the IPE 2 g/day and placebo groups. Significant placebo-adjusted decreases in the end points of TG, non–HDL-C, VLDL-C, Lp-PLA_2_, Apo B, TC, HDL-C, VLDL-TG, Ox-LDL, and RLP-C were observed in patients treated with IPE 4 g/day in all diabetes subgroups (Figure 1A; Table 1). Although placebo-adjusted reductions in hsCRP were not significant in the better-controlled diabetes group, significant placebo-adjusted reductions were achieved in the total and less-controlled diabetes subgroups (Figure 1A; Table 1). Effects were numerically more pronounced in patients with less-controlled diabetes for non–HDL-C, VLDL-C, Apo B, TC, VLDL-TG, hsCRP, and Ox-LDL compared with patients with better-controlled diabetes (Figure 1A; Table 1). In general, the magnitude of the reductions observed in patients receiving IPE 2 g/day was numerically lower than that observed with IPE 4 g/day (Figure 1B; Table 1). Furthermore, patients with less-controlled diabetes showed no significant placebo-adjusted reductions for any parameter following treatment with IPE 2 g/day. No statistically significant placebo-adjusted increases in LDL-C were observed in any subgroup with either dose of IPE. Notably, IPE 4 g/day significantly reduced placebo-adjusted LDL-C by 6.3% in the population of all patients with diabetes (Figure 1A; Table 1).

**Table 1:** **Change in efficacy parameters following IPE treatment in diabetes subgroups by median baseline A**_**1c**_

**Parameter**	**All patients with diabetes mellitus**		**A**_**1c **_**<6.8%**		**A**_**1c **_**≥6.8%**		**All patients with diabetes mellitus**	**A**_**1c **_**<6.8%**	**A**_**1c **_**≥6.8%**
**IPE dose (n = all patients, A**_**1c **_**<6.8%, A**_**1c **_**≥6.8%)**	**Baseline**	**End of treatment**	**Baseline**	**End of treatment**	**Baseline**	**End of treatment**	**Median placebo-adjusted change from baseline, %, *p***		
**TG (mg/dL)**									
4 g/day	262	217	262	228	268	203	-23.2	-21.0	-24.8
(n=165, 78, 87)	(220-312)	(179-267)	(223-316)	(186-290)	(218-309)	(166-261)	<0.0001	<0.0001	<0.0001
2 g/day	254	244	264	238	250	248	-9.8	-15.1	-4.8
(n=171, 81, 90)	(216-303)	(199-315)	(218-325)	(196-307)	(214-297)	(203-318)	<0.01	<0.01	0.39
Placebo	259	276	258	276	260	274			
(n=165, 87, 78)	(221-299)	(214-367)	(220-294)	(210-343)	(223-315)	(216-379)			
**LDL-C (mg/dL)**									
4 g/day	81	83	85	83	79	83	-6.3	-6.6	-5.7
(n=165, 78, 87)	(70-96)	(69-98)	(70-101)	(69-96)	(70-93)	(69-100)	0.02	0.08	0.13
2 g/day	82	87	83	89	82	85	-3.8	-3.7	-4.7
(n=170, 80, 90)	(73-97)	(74-100)	(76-97)	(78-99)	(69-96)	(72-102)	0.15	0.33	0.25
Placebo	84	88	84	85	84	93			
(n=164, 87, 77)	(72-97)	(74-105)	(70-97)	(75-102)	(72-97)	(72-108)			
**Non–HDL-C (mg/dL)**									
4 g/day	128	121	129	124	128	119	-14.4	-11.3	-18.0
(n=165, 78, 87)	(111-146)	(104-144)	(111-146)	(107-144)	(111-146)	(101-143)	<0.0001	<0.01	<0.0001
2 g/day	125	135	128	135	124	136	-4.4	-4.6	-4.2
(n=171, 81, 90)	(113-146)	(117-158)	(115-145)	(119-154)	(111-148)	(113-162)	0.07	0.17	0.22
Placebo	128	136	128	136	130	140			
(n=165, 87, 78)	(113-147)	(123-167)	(114-142)	(121-161)	(112-154)	(126-173)			
**VLDL-C (mg/dL)**									
4 g/day	44	37	45	41	43	35	-24.1	-14.3	-33.8
(n=165, 78, 87)	(33-54)	(29-50)	(32-53)	(31-52)	(33-56)	(28-47)	<0.0001	0.01	<0.0001
2 g/day	42	42	42	44	42	42	-6.1	-3.8	-9.0
(n=170, 80, 90)	(32-51)	(33-58)	(32-53)	(30-60)	(33-51)	(34-55)	0.21	0.57	0.17
Placebo	42	49	42	45	43	53			
(n=164, 87, 77)	(35-56)	(37-66)	(35-56)	(37-63)	(36-54)	(40-67)			
**Lp-PLA**_**2 **_**(ng/mL)**									
4 g/day	180	160	179	166	180	153	-19.1	-17.9	-20.3
(n=160, 75, 85)	(160-212)	(134-186)	(161-213)	(137-191)	(153-211)	(130-179)	<0.0001	<0.0001	<0.0001
2 g/day	192	184	196	185	182	178	-7.5	-9.5	-5.7
(n=163, 78, 85)	(164-219)	(159-215)	(169-237)	(163-219)	(161-212)	(153-210)	<0.001	<0.01	0.08
Placebo	184	197	189	200	181	188			
(n=156, 85, 71)	(159-219)	(165-233)	(166-221)	(170-241)	(142-216)	(163-220)			
**Apo B (mg/dL**)									
4 g/day	91	89	91	90	91	88	-9.5	-6.1	-12.8
(n=160, 75, 85)	(80-104)	(79-105)	(82-107)	(80-107)	(79-101)	(78-103)	<0.0001	0.02	<0.0001
2 g/day	91	95	91	96	91	95	-3.4	-2.1	-5.1
(n=166, 79, 87)	(84-104)	(86-107)	(84-105)	(87-106)	(83-103)	(85-112)	0.08	0.46	0.07
Placebo	93	98	93	96	93	103			
(n=158, 86, 72)	(82-105)	(88-113)	(82-104)	(86-109)	(82-106)	(90-117)			
**TC (mg/dL)**									
4 g/day	167	160	173	166	165	157	-12.7	-10.7	-15.1
(n=165, 78, 87)	(147-188)	(142-181)	(150-189)	(143-184)	(145-187)	(140-177)	<0.0001	0.0001	<0.0001
2 g/day	166	176	170	177	164	175	-4.0	-4.1	-4.1
(n=171, 81, 90)	(150-185)	(154-198)	(156-184)	(162-196)	(146-185)	(151-201)	0.04	0.10	0.15
Placebo	167	181	167	181	168	184			
(n=165, 87, 78)	(151-190)	(166-211)	(154-189)	(168-206)	(147-191)	(160-216)			
**HDL-C (mg/dL)**									
4 g/day	36	36	38	38	36	35	-5.0	-5.9	-4.3
(n=165, 78, 87)	(31-43)	(30-43)	(32-45)	(30-43)	(30-42)	(30-44)	<0.01	0.02	0.05
2 g/day	39	40	40	41	39	39	-2.3	-1.6	-2.9
(n=171, 81, 90)	(32-46)	(33-45)	(32-46)	(34-47)	(31-45)	(32-45)	0.17	0.51	0.18
Placebo	38	39	41	40	37	38			
(n=165, 87, 78)	(32-45)	(34-48)	(33-47)	(34-50)	(32-42)	(32-47)			
**VLDL-TG (mg/dL)**									
4 g/day	186	145	189	163	185	140	-28.9	-23.5	-34.1
(n=165, 78, 87)	(147-245)	(113-200)	(147-238)	(111-214)	(140-249)	(113-177)	<0.0001	<0.001	<0.0001
2 g/day	181	168	186	172	177	163	-10.8	-10.9	-10.7
(n=170, 80, 90)	(143-232)	(136-236)	(145-243)	(136-239)	(143-230)	(136-236)	0.02	0.12	0.11
Placebo	186	202	176	196	197	209			
(n=164, 87, 77)	(145-238)	(146-277)	(142-224)	(142-275)	(146-243)	(146-283)			
**Ox-LDL (U/L)**									
4 g/day	54	52	55	52	52	53	-16.1	-13.8	-17.4
(n=60, 29, 31)	(46-60)	(44-59)	(49-58)	(44-63)	(44-66)	(45-59)	<0.0001	<0.01	<0.001
2 g/day	55	56	55	57	53	56	-8.9	-10.3	-8.2
(n=59, 24, 35)	(48-66)	(49-71)	(49-59)	(49-62)	(48-69)	(49-74)	0.03	0.07	0.15
Placebo	52	60	52	60	53	61			
(n=64, 34, 30)	(45-62)	(51-70)	(46-63)	(51-66)	(42-62)	(47-73)			
**hsCRP (mg/L)**									
4 g/day	2.5	2.1	1.8	2.0	2.9	2.1	-21.5	-4.0	-34.6
(n=160, 75, 85)	(1.3-4.1)	(1.0-4.4)	(1.1-3.6)	(0.9-4.4)	(1.4-4.9)	(1.1-4.3)	<0.01	0.74	<0.001
2 g/day	2.1	2.7	1.5	1.7	2.5	3.2	-8.2	-8.4	-8.7
(n=166, 79, 87)	(1.0-4.6)	(1.1-4.5)	(0.9-3.1)	(0.9-3.9)	(1.3-5.8)	(1.5-5.0)	0.26	0.41	0.41
Placebo	2.5	3.1	2.1	2.7	2.7	3.4			
(n=158, 86, 72)	(1.2-5.1)	(1.3-6.0)	(1.1-5.0)	(1.2-6.1)	(1.4-5.2)	(1.4-5.9)			
**RLP-C (mg/dL)**									
4 g/day	13.0	10.0	14.0	11.5	12.5	9.0	-25.0	-26.7	-21.3
(n=60, 28, 32)	(11.0-17.0)	(8.0-13.6)	(11.0-16.6)	(8.0-14.5)	(10.0-18.0)	(8.0-11.5)	<0.01	0.05	0.04
2 g/day	15.0	11.0	16.0	13.0	12.0	10.5	-16.6	-27.8	-3.0
(n=62, 32, 30)	(11.0-18.0)	(9.0-16.0)	(13.0-18.5)	(10.0-16.0)	(10.0-18.0)	(8.0-14.0)	0.06	0.02	0.84
Placebo	14.0	13.0	14.0	15.5	14.0	12.0			
(n=61, 34, 27)	(11.0-17.0)	(9.0-20.0)	(11.0-18.0)	(11.0-22.0)	(11.0-16.0)	(9.0-16.0)			

Data are presented as median (lower and upper quartile) for end point values.

Apo B = apolipoprotein B; HDL-C = high-density lipoprotein cholesterol; hsCRP = high-sensitivity C-reactive protein; IPE = icosapent ethyl; LDL-C = low-density lipoprotein cholesterol; Lp-PLA_2_ = lipoprotein-associated phospholipase A_2_; non–HDL-C = non–high-density lipoprotein cholesterol; Ox-LDL = oxidized low-density lipoprotein; RLP-C = remnant-like particle cholesterol; TC = total cholesterol; TG = triglyceride; VLDL-C = very-low-density lipoprotein cholesterol; VLDL-TG = very-low-density lipoprotein triglycerides.

The mean baseline diabetes data in each diabetes subgroup is shown in Table 2. Baseline values for FPG were numerically higher in those with less-controlled diabetes than those with better-controlled diabetes. In each of the diabetes subgroups, no statistically significant placebo-adjusted changes were observed in the diabetes end points of FPG, A_1c_, HOMA-IR, or insulin following treatment with IPE 4 g/day or 2 g/day (Table 2).

**Table 2:** **Change in diabetes end points following IPE treatment in diabetes subgroups by median baseline A**_**1c**_

**Parameter**	**All patients with diabetes mellitus**		**A**_**1c **_**<6.8%**		**A**_**1c **_**≥6.8%**		**All patients with diabetes mellitus**	**A**_**1c **_**<6.8%**	**A**_**1c **_**≥6.8%**
**IPE dose (n=all patients, A**_**1c **_**<6.8%, A**_**1c**_ ≥6.8%)									
	**Baseline**	**End of treatment**	**Baseline**	**End of treatment**	**Baseline**	**End of treatment**	**Placebo-adjusted change from baseline, %, *p***		
**FPG, mg/dL**									
4 g/day	143	154	122	133	161	172	3.7	2.7	4.0
(n=160, 75, 85)	(38)	(53)	(23)	(37)	(39)	(59)	0.16	0.38	0.35
2 g/day	146	150	123	127	167	171	0.1	-2.8	2.6
(n=165, 79, 86)	(44)	(47)	(25)	(26)	(47)	(51)	0.99	0.36	0.54
Placebo	140	145	124	130	158	163			
(n=158, 86, 72)	(35)	(39)	(25)	(30)	(37)	(41)			
**A**_**1c**_**, %**									
4 g/day	6.9	7.2	6.2	6.5	7.6	7.9	1.4	1.3	1.7
(n=161, 76, 85)	(0.9)	(1.1)	(0.3)	(0.6)	(0.7)	(1.1)	0.14	0.27	0.29
2 g/day	7.0	7.2	6.2	6.3	7.8	8.0	0.1	-0.4	0.8
(n=167, 79, 88)	(1.1)	(1.2)	(0.4)	(0.5)	(0.9)	(1.1)	0.88	0.71	0.59
Placebo	6.8	7.0	6.2	6.4	7.5	7.7			
(n=158, 86, 72)	(0.9)	(1.1)	(0.4)	(0.7)	(0.7)	(0.9)			
**Insulin, μIU/mL**									
4 g/day	20.6	18.5	20.0	19.3	21.2	17.7	-5.9	0.6	-13.4
(n=159, 76, 83)	(16.0)	(10.6)	(12.1)	(9.8)	(19.0)	(11.3)	0.43	0.95	0.26
2 g/day	18.9	19.1	16.8	17.4	21.1	20.7	-4.1	9.2	-17.8
(n=157, 79, 78)	(11.6)	(10.9)	(9.3)	(9.0)	(13.2)	(12.5)	0.59	0.32	0.14
Placebo	25.4	21.5	27.5	19.6	22.8	23.8			
(n=156, 85, 71)	(38.2)	(18.9)	(48.2)	(13.9)	(20.6)	(23.5)			
**HOMA-IR**									
4 g/day	7.5	7.3	6.1	6.4	8.8	8.1	-1.4	3.0	-7.1
(n=158, 75, 83)	(7.5)	(6.2)	(3.8)	(3.7)	(9.5)	(7.7)	0.88	0.79	0.64
2 g/day	7.0	7.1	5.2	5.5	8.8	8.7	-8.8	6.0	-23.9
(n=157, 79, 78)	(5.0)	(4.8)	(3.4)	(3.1)	(5.6)	(5.6)	0.36	0.60	0.13
Placebo	9.6	7.9	9.7	6.4	9.4	9.6			
(n=156, 85, 71)	(19.0)	(7.6)	(24.1)	(5.0)	(9.9)	(9.6)			

Mean and standard deviation (SD) are reported for baseline and end-of-treatment values. Least-squares means are reported for the placebo-adjusted changes from baseline.

A_1c_ = glycosylated hemoglobin A_1c_; FPG = fasting plasma glucose; HOMA-IR = homeostasis model assessment-estimated insulin resistance; IPE = icosapent ethyl.

## Discussion

In this subanalysis of the ANCHOR study, treatment with IPE (a high-purity prescription form of EPA ethyl ester) at a dose of 4 g/day was shown to significantly reduce TG without raising LDL-C in a cohort of statin-treated patients with diabetes mellitus and residually high TG levels (≥200 and <500 mg/dL) despite LDL-C control (≥40 and <100 mg/dL). In the ANCHOR study, treatment with IPE 4 g/day resulted in numerically greater reductions in patients with diabetes mellitus compared with patients with no diabetes mellitus [10]. In addition to improving TG levels in patients with diabetes mellitus, this analysis found that, compared with placebo, treatment with IPE 4 g/day also significantly decreased placebo-adjusted non–HDL-C, VLDL-C, Lp-PLA_2_, Apo B, TC, HDL-C, VLDL-TG, and hsCRP. These results are consistent with those reported for the overall ANCHOR population [10]. In this analysis, Ox-LDL and RLP-C were also significantly decreased by treatment with IPE 4 g/day. Of particular interest, the decreases in non–HDL-C, VLDL-C, Apo B, TC, VLDL-TG, hsCRP, and Ox-LDL were numerically more pronounced in patients who had less-controlled diabetes at baseline compared with patients who had better-controlled diabetes at baseline.

The TG-lowering effects of OM-3 fatty acids are well established, have been demonstrated in patients with diabetes mellitus [15,16], and are supported by this subanalysis of the ANCHOR study. Significant reductions in TC, LDL-C, and non–HDL-C have also recently been reported in a small study of patients with diabetes mellitus-2 and dyslipidemia following treatment with 1.8 g/day of purified EPA [17]. However, in another small study of patients with diabetes mellitus and comorbid major depressive disorder, 1.0 g/day of >90% pure EPA ethyl ester resulted in small but significant increases in TC and HDL-C but not in LDL-C [18]. The dose was notably low at 1 g/day and there were differences in tocopherol concentrations between the EPA intervention and placebo formulations. As with the present analysis, reductions in VLDL-C and VLDL-TG were also reported in a systematic meta-analysis by Hartweg et al. [15] of patients with diabetes mellitus-2 from studies wherein most patients were treated with EPA plus DHA. However, modest increases in LDL-C were noted in the meta-analysis [15] while this subanalysis found no significant change in LDL-C following treatment with IPE 2 g/day and a statistically significant decrease of 6.3% in LDL-C with IPE 4 g/day compared with placebo in the population of all patients with diabetes (*P* = 0.02). This ability of a pure EPA product (such as IPE) to reduce LDL-C, in contrast with pure DHA or DHA/EPA combinations, which tend to raise LDL-C, is supported by 2 recent meta-analyses, which found that while DHA raised LDL-C, EPA did not [19,20].

The potentially favorable decreases in TG, non–HDL-C, VLDL-C, Apo B, hsCRP, Ox-LDL, and RLP-C levels seen with IPE compared with placebo may be of clinical importance because these parameters are more likely to be abnormal in patients with diabetes mellitus (especially when less controlled), and it is believed that increases in lipid, lipoprotein, and inflammatory end points may contribute to the excess CVD risk in this setting [5]. Glycosylated hemoglobin has been shown to be significantly and positively associated with elevated concentrations of CRP [21]. In this study, baseline hsCRP was numerically higher in patients with less-controlled diabetes mellitus than in those with better-controlled diabetes mellitus and thus may be indicative of greater inflammation in patients with less-controlled diabetes mellitus [22]. The reductions observed in RLP-C in the present study may be clinically important because remnant lipoproteins may be atherogenic [23,24]. The finding that IPE does not increase (and may modestly lower) LDL-C suggests a potential clinical benefit of IPE in that it may not interfere with reaching or maintaining LDL-C goal in patients with diabetes mellitus.

The clinical importance of a modest decrease of 5% in HDL-C observed in this study with the 4 g/day dose compared with placebo is unclear. This finding, however, is the opposite of the modest increases in HDL-C often shown with other OM-3 fatty acid treatments, especially those with substantial DHA content, which is absent in IPE [25,26]. Interestingly, an independent measure of HDL concentration provided by nuclear magnetic resonance (HDL particle concentration, HDL-P) has been reported to decrease with both EPA-only [27] and combination EPA/DHA OM-3 fatty acid preparations [28]. Furthermore, based on the results of 2 recent CVD outcomes trials, AIM-HIGH and dal-OUTCOMES, the role of changes in HDL-C levels as a reliable inverse predictor of changes in CVD risk has been called into question [29,30].

No statistically significant changes in glycemic control were observed following treatment with IPE 4 g/day or 2 g/day compared with placebo. This absence of observed increases in glycemic measures may be of clinical importance, since a prior study showed a statistically significant increase in FPG following treatment with a prescription formulation of EPA plus DHA [26]. In contrast, a recent systematic review and meta-analysis found that consumption of EPA and/or DHA did not alter the risk of the development of diabetes mellitus [31].

A limitation of this study is the fact that all of the diabetes subgroup analyses were performed post hoc with the exception of TG levels, which was prespecified. Furthermore, the effect of the reductions in lipid and inflammatory parameters reported here and elsewhere on CVD risk with OM-3 fatty acid treatment is not yet known, particularly in patients with diabetes mellitus. However, the American Diabetes Association recommends that OM-3 fatty acid consumption in patients with diabetes be increased by consuming ≥2 servings of fish per week [32], and evidence has begun to accrue, in subanalyses of OM-3 fatty acid levels in several observational CVD outcomes studies in patients with diabetes mellitus, that higher plasma OM-3 levels predict reduced CVD risk [33-39].

Supplementation with 1 g/day OM-3 fatty acids was found to reduce total mortality in patients with recent myocardial infarction in the Gruppo Italiano per lo Studio della Sopravvivenza nell’Infarto miocardico-Prevenzione trial (GISSI-Prevenzione) [33,34]. Approximately 15% of the GISSI-Prevenzione patients had diabetes mellitus, and a retrospective subgroup analysis found that the efficacy of OM-3 fatty acid administration on total mortality was similar in the absence and presence of diabetes mellitus [35]. Secondary and post hoc exploratory analyses of patients with diabetes from the Alpha Omega Trial demonstrated reductions in certain cardiovascular end points with low-dose EPA plus DHA treatment [36,37]. In a subanalysis of the Japan EPA Lipid Intervention Study (JELIS), purified EPA was found to be very effective in reducing the incidence of coronary artery disease in patients with impaired glucose metabolism [39].

Furthermore, a recent review of clinical studies in heart failure patients found that OM-3 fatty acids may have preferential beneficial therapeutic effects in patients with diabetes mellitus [38]. This is consistent with data from a rodent model of type 2 diabetes mellitus, in which myocardial content EPA and DHA was lower in rats with type 2 diabetes mellitus and may therefore be a factor associated with cardiac pathophysiology in type 2 diabetes mellitus [40].

In contrast to these positive results, recent results from the Outcome Reduction with Initial Glargine Intervention (ORIGIN) study found that treatment with EPA plus DHA did not prevent death or any cardiovascular outcomes in patients with increased risk of cardiovascular events who had diabetes mellitus or who were at high risk for diabetes mellitus [41]. A potential explanation for the discrepancy in results between ORIGIN and JELIS is that the dose of EPA, or EPA plus DHA, in JELIS was 1.8 g/day, approximately twice that of ORIGIN (and indeed most of the earlier studies).

The ongoing Reduction of Cardiovascular Events with EPA-Intervention (REDUCE-IT; NCT1492361) study will evaluate the effect of IPE 4 g/day on prevention of a first major cardiovascular event in approximately 8000 patients with hypertriglyceridemia at high risk for cardiovascular events, including a large cohort of patients with diabetes mellitus. Results from REDUCE-IT promise to provide crucial information regarding the utility of IPE for therapy in patients at risk for CVD, including patients with diabetes mellitus and associated dyslipidemia.

## Conclusions

IPE 4 g/day significantly improved most major lipid and lipoprotein parameters (including TG-rich remnant lipoproteins) and inflammatory parameters without worsening LDL-C or glycemic control in patients with diabetes and mixed dyslipidemia. Many of these effects appeared to be greater among those with less-controlled diabetes, suggesting that IPE may be especially beneficial in patients with diabetes when optimal glycemic control has not been or cannot be achieved.

## Abbreviations

A1c: Glycosylated hemoglobin A_1c_; Apo B: Apolipoprotein B; CHD: Coronary heart disease; CVD: Cardiovascular disease; DHA: Docosahexaenoic acid; EPA: Eicosapentaenoic acid; FPG: Fasting plasma glucose; GISSI-Prevenzione: Gruppo Italiano per lo Studio della Sopravvivenza nell’Infarto miocardico-Prevenzione trial; HDL-C: High-density lipoprotein cholesterol; HOMA-IR: Homeostasis model assessment-estimated insulin resistance; hsCRP: High-sensitivity C-reactive protein; IPE: Icosapent ethyl; ITT: Intent-to-treat; JELIS: Japan EPA Lipid Intervention Study; LDL-C: Low-density lipoprotein cholesterol; Lp-PLA2: Lipoprotein-associated phospholipase A_2_; NCEP-ATP III: National Cholesterol Education Program Adult Treatment Panel III; OM-3: Omega-3; ORIGIN: Outcome Reduction with Initial Glargine Intervention study; Ox-LDL: Oxidized low-density lipoprotein; REDUCE-IT: Reduction of Cardiovascular Events with EPA-Intervention study; RLP-C: Remnant-like particle cholesterol; TC: Total cholesterol; TG: Triglycerides; VLDL-C: Very-low-density lipoprotein cholesterol; VLDL-TG: Very-low-density lipoprotein triglycerides.

## Competing interests

EAB has received grant/research support from Amarin Pharma Inc., Health Diagnostic Laboratory, and Roche; serves on the speakers bureaus (including receipt of honoraria) for Abbott, BMS, Boehringer, Daiichi-Sankyo, Kowa, Merck, and Takeda; provides consultancy services (including receipt of honoraria) for Abbott, Amarin Pharma Inc., Atherotech, Essentialis, Kowa, and Merck. CMB has received grant/research support from Abbott, AstraZeneca, BMS, diaDexus, GlaxoSmithKline, Kowa, Merck, Novartis, Roche, Sanofi-Synthelabo, Takeda, NIH, ADA, and AHA; has received speakers bureau fees from Abbott, GlaxoSmithKline, and Merck; has received honoraria from Abbott, AstraZeneca, GlaxoSmithKline, Merck, Sanofi-Synthelabo, and Takeda; and has provided consultancy services for Abbott, Adnexus, Amarin Pharma Inc., Amylin, AstraZeneca, BMS, Esperion, Genentech, GlaxoSmithKline, Idera Pharma, Kowa, Novartis, Omthera, Resverlogix, Roche, Sanofi-Synthelabo, and Takeda. HEB’s research site has received research grants from Amarin Pharma Inc., Amgen, Ardea, Arena, Boehringer Ingelheim, Cargill Inc., California Raisin Board, Esperion, Essentialis, Forest Laboratories, Gilead Sciences Inc., Given, GlaxoSmithKline, High Point Pharmaceuticals, Hoffman LaRoche, Home Access, Johnson and Johnson, Merck & Co., Micropharma, Novartis, Novo Nordisk, Omthera, Orexigen, Pfizer Inc., Pozen, Proctor and Gamble, Shionogi, Stratun Nutrition, Takeda Pharmaceuticals, TransTech Pharma, Trygg Pharmaceuticals, TWI Bio, Vivus, WPU, and Xoma. HEB has received consulting fees or speaking honoraria from Amgen, Amarin Pharma Inc., Bristol-Myers Squibb, Daiichi Sankyo, Eisai, Merck & Co., NovoNordisk, and Vivus. JJK serves on the speakers bureaus of Merck Sharp & Dohme, Pfizer, Roche, and AstraZeneca and is a consultant for Amarin Pharma Inc., Merck Sharp & Dohme, Roche, and AstraZeneca. RAB and PNS are employees and stock shareholders of Amarin Pharma Inc.

## Authors’ contributions

RAB and PNS were responsible for the study concept and design. RAB was responsible for acquisition of data. All authors were responsible for analysis and interpretation of data, drafting the manuscript, critical revision of the manuscript for important intellectual content, and final approval of the manuscript. RAB was responsible for statistical analysis. PNS obtained funding. RAB and PNS were responsible for administrative, technical, and material support. RAB, PNS, and JJK (Europe) were responsible for study supervision. All authors read and approved the final manuscript.
